# Hemophagocytic lymphohistiocytosis in adults: collaborative analysis of 137 cases of a nationwide German registry

**DOI:** 10.1007/s00432-020-03139-4

**Published:** 2020-02-20

**Authors:** Sebastian Birndt, Thomas Schenk, Babett Heinevetter, Frank M. Brunkhorst, Georg Maschmeyer, Frank Rothmann, Thomas Weber, Markus Müller, Jens Panse, Olaf Penack, Roland Schroers, Jan Braess, Norbert Frickhofen, Stephan Ehl, Gritta Janka, Kai Lehmberg, Mathias W. Pletz, Andreas Hochhaus, Thomas Ernst, Paul La Rosée

**Affiliations:** 1 Klinik für Innere Medizin II, Abt. Hämatologie und Intern. Onkologie, Universitätsklinikum Jena, Jena, Germany; 2 Zentrum für Klinische Studien, Universitätsklinikum Jena, Jena, Germany; 3 Klinik für Hämatologie, Onkologie u. Palliativmedizin, Klinikum Ernst Von Bergmann, Potsdam, Germany; 4 Klinik für Hämatologie und Onkologie, Universitätsklinikum Halle (Saale), Halle, Germany; 5 Zentrum für Infektiologie und HIV, Vivantes Auguste-Viktoria-Klinikum, Berlin, Germany; 6 Klinik für Hämatologie, Onkologie, Hämostaseologie und Stammzelltransplantation, Uniklinik RWTH Aachen, Aachen, Germany; 7 Medizinische Klinik mit Schwerpunkt Hämatologie, Onkologie und Tumorimmunologie, Charité Universitätsmedizin, Berlin, Germany; 8 Hämatologie und Onkologie, Universitätsklinikum Knappschaftskrankenhaus, Bochum, Germany; 9 Onkologie und Hämatologie, Krankenhaus Barmherzige Brüder, Regensburg, Germany; 10 Hämatologie, Onkologie und Palliativmedizin, HELIOS Dr. Horst Schmidt Kliniken, Wiesbaden, Germany; 11 Centrum für Chronische Immundefizienz (CCI), Universitätsklinikum Freiburg, Freiburg, Germany; 12 Pädiatrische Hämatologie und Onkologie, Universitätsklinikum Eppendorf, Hamburg, Germany; 13 Institut für Infektionsmedizin und Krankenhaushygiene, Universitätsklinikum Jena, Jena, Germany; 14 Klinik für Innere Medizin II, Hämatologie, Onkologie, Immunologie, Infektiologie und Palliativmedizin, Schwarzwald-Baar Klinikum, Klinikstr. 11, 78052 Villingen-Schwenningen, Germany

**Keywords:** HLH, Hemophagocytic lymphohistiocytosis, Sepsis, Inflammation, Cytokine storm

## Abstract

**Purpose:**

Hemophagocytic lymphohistiocytosis (HLH) is a severe hyperinflammatory syndrome emerging from a deregulated immune response due to various triggers. In adults, systematic data are sparse, which is why recommendations on diagnosis and management have been adopted from pediatric guidelines. A nationwide clinical registry with associated consulting service as collaborative initiative of HLH-specialized pediatricians and hematologists was initiated to better characterize HLH in adults.

**Methods:**

Patients with proven or suspected HLH were registered by 44 institutions. Both HLH-2004 diagnostic criteria and the HScore (www.saintantoine.aphp.fr/score/) were used to confirm HLH diagnosis. Data referring to underlying disease, treatment, outcome, clinical presentation and laboratory findings were recorded.

**Results:**

The study included 137 patients and provides the first systematic data on adult HLH in Germany. Median age was 50 years with a wide range (17–87 years), 87 patients (63.5%) were male. Most common triggering diseases were infections in 61 patients (44.5%) and malignancies in 48 patients (35%). Virtually all patients had elevated ferritin concentrations, and 74% had peak concentrations greater than 10,000 µg/l. At time of analysis, 67 of 131 patients (51%) had died. Patients with malignancy-associated HLH had the shortest median survival (160 days), however no statistically significant difference between subgroups was observed (*p* = 0.077). Platelets under 20*10^9^/l and low albumin concentrations (< 20 g/l) were associated with poor overall and 30-day survival.

**Conclusion:**

Close multidisciplinary case consultation and cooperation is mandatory when treating adult HLH patients. Early contact with reference centers is recommended, especially in relapsing or refractory disease.

**Supplementary Information:**

The online version contains supplementary material available at 10.1007/s00432-020-03139-4.

## Introduction

Hemophagocytic lymphohistiocytosis (HLH) is a hyperinflammatory syndrome driven by excessive activation and stimulation of cytotoxic T-lymphocytes, natural killer T-cells and macrophages with subsequent cytokine storm and organ damage (Janka and Lehmberg 2014). In adults, this often fatal aberrant immune response most frequently is triggered by infections and malignancies, or a combination of these. Patients with long-term immunosuppression are at increased risk to develop HLH (Ramos-Casals et al. 2014), as are patients with autoimmune/autoinflammatory disorders. By convention, and with impact on differential treatment, HLH in patients with autoimmune/autoinflammatory diseases is also called macrophage activation syndrome (MAS-HLH) (Emile et al. 2016). The wide spectrum of HLH-initiating conditions in adults is reflected by the term “acquired” or “secondary” HLH.

In contrast, primary HLH typically manifests in childhood, often has a family history, and is linked to mutations in genes involved in lymphocyte cytotoxicity. This includes PRF1, coding for perforin, or genes involved in the transport or exocytosis of perforin-containing lytic granules (Sepulveda and de Saint Basile 2017). Immunodeficiency syndromes, commonly associated with albinism, also predispose to HLH (Henkes et al. 2015). Clinically, a triad consisting of prolonged fever, hepatosplenomegaly and pancytopenia is common. However, a number of endogenous (i.e., genetic predisposition, preexisting inflammation) and exogenous factors (i.e., immunosuppression, triggering disease) play a role in HLH pathogenesis (Brisse et al. 2016). The spectrum of possible underlying conditions and patient characteristics is reflected by distinct clinical presentations which also can mimic other diseases, making timely diagnosis challenging. Specifically, HLH often is indistinguishable from sepsis or autoinflammatory diseases (e.g., adult-onset Still disease). As a result, and despite a high index of suspicion, there is high likelihood for mis- or underdiagnosing HLH, especially in intensive care units (Lachmann et al. 2018). HLH diagnosis is based on the HLH-2004 diagnostic criteria established by the Histiocyte Society (summarized in Table 1) (Henter et al. 2007). Of note, these criteria were established in the pediatric setting along the HLH-1994 and HLH-2004 trial protocols including patients up to 18 years (Bergsten et al. 2017; Trottestam et al. 2011). According to HLH-2004 study criteria, HLH can be diagnosed in a patient with at least 5 of 8 diagnostic criteria and/or disease-causing mutations in HLH-related genes. Recently, adaptation of diagnostic criteria has been proposed by French investigators, considering the impact of graded parameters and of state of immune competence on diagnostic accuracy (Fardet et al. 2014). The diagnostic algorithm is available as web-based tool to calculate HLH probability (https://saintantoine.aphp.fr/score/).

**Table 1:** HLH-2004 diagnostic criteria according to (Henter et al. 2007)

At least one of either (1) or (2) must be fulfilled:	
(1) Molecular diagnosis consistent with HLH	
(2) At least 5 of the 8 following criteria:	
a. Fever	
b. Cytopenia of two or more lineages	Hemoglobin < 90 g/l, ANC < 1 × 10^9^/l, Platelets < 100 × 10^9^/l
c. Splenomegaly	
d. Hypertriglyceridemia and/or hypofibrinogenemia	Fasting triglycerides ≥ 3 mmol/l Fibrinogen < 1.5 g/l
e. Hyperferritinemia	Ferritin ≥ 500 µg/l
f. Elevated sIL-2R (sCD25)	sIL-2R ≥ 2400 U/ml
g. Low or absent NK-cell activity	
h. Hemophagocytosis in bone marrow, spleen, or lymph node	

*ANC* absolute neutrophil count, *sIL-2R* soluble interleukin-2 receptor, *NK-cell* natural killer cell

Since HLH in adults is a rare and most probably underreported syndrome, our current knowledge relies on case reports and series (Hayden et al. 2016; Ramos-Casals et al. 2014). Therefore, a clinical registry was initiated, aiming to better characterize and understand the spectrum of triggering conditions and management of adult HLH in Germany. Screening and inclusion of patients was based on a clinical consulting service that was made public by the Onkopedia platform HLH-guideline (www.onkopedia.com) and via www.hlh-registry.org.

In this report, the first analysis of the registry including 137 patients  ≥ 17 years is presented. A special focus was put on underlying diseases, clinical and laboratory findings, and potential prognostic factors.

## Patients and Methods

This registry for adult hemophagocytic lymphohistiocytosis was launched in August 2010, with the aim to collect data on epidemiology, treatment, clinical and laboratory characteristics, and outcome of affected patients. Data collection was based on a clinical consulting service for adult patients with suspected or proven HLH. At the time of analysis (June 30th 2017), 156 patients with suspected or proven HLH had been enrolled by 44 medical institutions. Patients with confirmed HLH were reported primarily from hematology/oncology centers (38/44 centers). After informed consent, an online documentation form (available at www.hlh-registry.org) was used for initial data submission. In addition, medical records were reviewed to obtain further information on clinical course and treatment. Anonymized patient data were subsequently recorded in an online-based OpenClinica database (Waltham, MA, US).

To confirm HLH diagnosis, patients were evaluated using both the HLH-2004 criteria (Table 1) and the HScore (supplementary Table 1, online available at: https://saintantoine.aphp.fr/score/) to quantify the probability of having HLH (Fardet et al. 2014). Of a total of 156 enrolled patients, 129 patients (82.7%) were eligible for analysis on the basis of HLH-2004 diagnostic criteria (i.e., molecular diagnosis and/or at least five out of eight HLH criteria). Furthermore, we included eight additional patients who met four diagnostic criteria and reached HLH-probability of more than 90% according to the HScore. Thus, 137 of 156 patients (88%) were suitable for further analysis. A flow chart illustrating our approach is provided in the supplement (supplementary Fig. 1 ). According to the most likely triggering disease based on medical information, patients were categorized into four different subgroups for either malignancy-associated HLH, infection-associated HLH, MAS-HLH, or HLH due to an unknown trigger. In 20 patients with available blood or bone marrow samples, targeted perforin sequencing was performed.

### Statistical analysis

Results are presented as median plus range, frequencies, or percentages. Overall survival was defined as time from date of diagnosis to date of death from any cause or date of last follow-up, respectively. Patients without available follow-up data were excluded from further analysis. Kaplan–Meier method was used to visualize median survival times, and the log-rank test was used to compare subgroups. Cox´s proportional hazards model was used for univariate and multivariate analyses. Variables with a *p* value < 0.05 in univariate analysis were included in multivariate analysis to determine independent predictive factors. A backward stepwise selection procedure (Wald) was performed, with significance level for exclusion set at 0.1. All statistical tests were two-sided. *p* values < 0.05 were considered statistically significant. Correlation between HLH-2004 diagnostic criteria and the HScore was analyzed using Pearson´s *r*. All statistical analyses were performed using IBM SPSS Version 24 (IBM Corp., Armonk, N.Y., US).

### Genetic analysis

Genomic DNA was isolated from peripheral blood samples or bone marrow according to manufacturer instructions using the QIAamp DNA Mini Kit (Qiagen, Hilden, Germany). Coding exons of the perforin gene (PRF1) were amplified by polymerase chain reaction (PCR). Sanger sequencing was performed using the ABI 3500 Genetic Analyzer (Thermo Fisher Scientific Inc., Waltham, MA, US), mutation analysis was done using Mutation Surveyor software (SoftGenetics LLC., State College, PA, US).

## Results

### Patient characteristics

Of 137 eligible patients, 50 patients were female (36.5%) and 87 patients were male (63.5%). Median age at diagnosis was 50 years, ranging from 17 to 87 years. Information on time to diagnosis from date of first symptoms was available for 124 patients, with a median of 10 days (range 1–93 days). In 27 patients (21.8%), HLH diagnosis was made later than 21 days after first presentation. At onset of HLH, preceding immunosuppression (i.e., azathioprine treatment, cyclosporine in patients after allogeneic stem cell transplantation) was present in 58 of 128 patients (45.3%). For all patients enrolled (*n* = 156), the HScore was calculated. There was a significant direct correlation between the HLH-2004 diagnostic criteria and the HScore (*R* = 0.75, *p* < 0.001), a scatter diagram is provided in Supplementary Fig. 2. One hundred and twenty-two of 137 patients (89%) eligible for further analysis had HScore values greater than 203 points, i.e., a probability for HLH of more than 90%.

## Triggering diseases

The most frequent triggering diseases in our cohort were infections (*n* = 61, 44.5% of all patients) and malignancies (*n* = 48, 35%). In malignancy-associated HLH, hematologic neoplasia, in particular lymphomas of B-lymphoid origin represented the main cause. Myeloid disorders, e.g., acute myeloid leukemia, were seen in a minority (*n* = 7, 5.1%). Active Herpes virus infections (i.e., evidence of viremia by PCR) such as EBV (*n* = 21, 15.3%) or CMV (*n* = 4, 2.9%) were most prevalent in infection-associated HLH. Six patients developed HLH due to viral infections after allogeneic stem cell transplantation (EBV, *n* = 5; CMV, *n* = 1). HIV infection was identified in 4 patients, of whom two had HIV/HHV8 co-infection. Bacterial infections were diagnosed in five patients (3.6%), while fungal infection was diagnosed in one patient. Visceral leishmaniasis was detected in three patients, of whom two had preceding immune-modulating treatments with adalimumab and tocilizumab, respectively. In 13 patients (9.5%) an infection was the likely triggering disease, as these patients presented with elevated procalcitonin and showed evidence for an infectious source (i.e., radiological evidence, infiltrates in radiography or CT scan), however no infectious agent was identified in this group. HLH due to autoimmune/autoinflammatory diseases (MAS-HLH) was diagnosed in 13 patients (9.5%), with adult-onset Still disease being predominant. In five patients, disease-causing mutations in HLH-related genes were identified. In 15 patients (10.9%), a trigger could not be identified. A detailed overview demonstrating the spectrum of etiologies is provided in Table 2.

**Table 2:** Underlying conditions

	*n* (%)
Malignant diseases	48 (35.0)
Myeloid	7 (5.1)
AML	3 (2.2)
CML	1 (0.7)
MDS/MPN overlap syndrome	3 (2.2)
B-Lymphoid	30 (21.9)
DLBCL	11 (8.0)
Intravascular large B-cell lymphoma	1 (0.7)
Hodgkin lymphoma	7 (5.1)
Mantle cell lymphoma	1 (0.7)
Marginal zone lymphoma	2 (1.5)
B-CLL	2 (1.5)
B-ALL	1 (0.7)
B-cell lymphoma (no further information)	5 (3.6)
T-lymphoid	10 (7.3)
Peripheral T-cell lymphoma	2 (1.5)
NK/T-cell lymphoma	2 (1.5)
T-ALL	1 (0.7)
NK-cell leukemia	1 (0.7)
Angioimmunoblastic T-cell lymphoma	2 (1.5)
Anaplastic large cell lymphoma	1 (0.7)
Enteropathy-associated T-cell lymphoma	1 (0.7)
Solid	1 (0.7)
Testicular-mixed tumor	1 (0.7)
Infections	61 (44.5)
Viral	39 (28.5)
EBV	21 (15.3)
EBV after allogeneic stem cell transplantation	5 (3.6)
CMV	4 (2.9)
CMV after allogeneic stem cell transplantation	1 (0.7)
CMV/EBV coinfection	3 (2.2)
H1N1/EBV coinfection	1 (0.7)
HHV6	1 (0.7)
HIV	2 (1.5)
HHV8/HIV coinfection	2 (1.5)
VZV	1 (0.7)
HSV	1 (0.7)
Influenza A	1 (0.7)
Parvovirus B19	1 (0.7)
Hantavirus	1 (0.7)
Bacterial	5 (3.6)
*Proteus* spp.	1 (0.7)
* Klebsiella pneumoniae*	1 (0.7)
* Salmonella typhii*	1 (0.7)
* Pseudomonas aeruginosa*	(0.7)
* Staphylococcus Epidermidis*	1 (0.7)
Fungal	1 (0.7)
* Histoplasma capsulatum*	1 (0.7)
Parasite	3 (2.2)
Leishmaniasis	3 (2.2)
Infection without documented pathogen	13 (9.5)
Autoimmune/inflammatory diseases	13 (9.5)
Adult-onset still disease	8 (5.8)
Rheumatoid arthritis	2 (1.5)
Systemic lupus erythematosus	1 (0.7)
Crohn´s disease	1 (0.7)
ANCA negative vasculitis	1 (0.7)
Idiopathic	15 (10.9)
Total	137

Of 14 patients with malignancy-associated HLH, 12 also had EBV replication, one patient had CMV replication, and one patient had EBV replication and HIV infection. Percentages may not add to 100 because of rounding

*AML* acute myeloid leukemia, *CML* chronic myeloid leukemia, MDS/MPN myelodysplastic/myeloproliferative neoplasm, *DLBCL* diffuse large B-cell lymphoma, *CLL* chronic lymphocytic leukemia, *ALL* acute lymphocytic leukemia, *EBV* epstein-Barr virus, *CMV* cytomegalovirus, *HHV* human herpes virus, *HIV* human immunodeficiency virus, *VZV* varicella zoster virus, *HSV* herpes simplex virus, *ANCA* anti-neutrophil cytoplasmic antibody

## Clinical presentation and laboratory findings

Clinical presentation in adult HLH included a variable combination of symptoms, though fever and splenomegaly were most common (in 98% and 86% of all patients, Table 3). 82 of 133 patients (62%) fulfilled the clinical triad consisting of fever, splenomegaly and cytopenia of at least two lineages. Other common clinical findings included hepatomegaly (61% of patients), renal failure (47%), pulmonary symptoms such as respiratory insufficiency (33%), and neurological symptoms (31%). Based on laboratory parameters such as albumin, bilirubin, and transaminases, virtually all patients had liver dysfunction or damage, while 32 patients presented with bleeding complications or disseminated intravascular coagulation (DIC). Laboratory characteristics are presented in Table 4. 73% of the patients had cytopenia of at least two hematopoietic lineages. Nearly all patients (99%) had increased serum ferritin concentrations, with a median peak concentration of 30,281 µg/l. One-hundred of 135 patients (74%) showed peak values above 10,000 µg/l. Extreme ferritin concentrations (> 50,000 µg/l) were found in 42 patients (31%). Elevated concentrations of fasting triglycerides and soluble interleukin-2 receptor (sIL-2R) were documented in 70 and 94% of patients, respectively. Decreased fibrinogen concentrations were present in 41% of the patients. Hemophagocytosis in either bone marrow samples (*n* = 79) or lymph nodes (*n* = 2) was detected in 81 of 129 patients (63%).

**Table 3:** Clinical manifestations

	Number of patients	(%)
Fever	134/137	98
Splenomegaly	115/133	86
Triad of fever, splenomegaly, cytopenia	82/133	62
Cytopenia (at least two lineages)*	99/135	73
Neutropenia (ANC < 1 × 10^9^/l)	51/119	43
Anemia (Hemoglobin < 90 g/l)	97/135	72
Thrombocytopenia (Platelets < 100 × 10^9^/l)	111/135	82
Hemophagocytosis (bone marrow, spleen, or lymph node)	81/129	63
Hepatomegaly	63/103	61
Renal involvement, acute renal failure	52/111	47
Neurological symptoms	41/131	31
Bleeding complications, manifest DIC	44/136	32
Pulmonary involvement, respiratory insufficiency	40/121	33
Peripheral lymphadenopathy	38/116	33

*ANC* absolute neutrophil count, *DIC* disseminated intravascular coagulation

*ANC < 1 × 10^9^/l; Hemoglobin < 90 g/l; Platelets < 100 × 10^9^/l

**Table 4:** Laboratory findings. If not specifically marked, all data are presented using frequencies; corresponding percentages are presented in parentheses

	Overall	M-HLH	I-HLH	MAS-HLH	Idiopathic
Number of patients	137	48	61	13	15
Median age in years (range)	50 (17–87)	60 (18–87)	43 (17–71)	40 (18–78)	55 (19–81)
Male (*n*)	87	32	40	7	8
Female (*n*)	50	16	21	6	7
HLH 2004 criteria					
Fever	134/137 (98)	47/48 (98)	60/61 (98)	13/13 (100)	14/15 (93)
Splenomegaly	115/133* (86)	42/45 (93)	48/60 (80)	12/13 (92)	13/15 (87)
Cytopenias (of at least two lineages)	99/135 (73)	42/48 (88)	44/59 (75)	5/13 (38)	8/15 (53)
Neutropenia (ANC < 1 × 10^9^/l)	51/119 (43)	18/42 (43)	23/50 (46)	3/12 (25)	7/15 (47)
Anemia (Hemoglobin < 90 g/l)	97/135 (72)	39/48 (81)	40/59 (68)	8/13 (62)	10/15 (67)
Thrombocytopenia (Platelets < 100 × 10^9^/l)	111/135 (82)	45/48 (94)	52/59 (88)	6/13 (46)	8/15 (53)
Hypertriglyceridemia (> 3 mmol/l)	86/123 (70)	36/44 (82)	35/54 (65)	8/12 (67)	7/13 (54)
Hypofibrinogenemia (< 1.5 g/l)	52/127 (41)	20/44 (45)	23/56 (41)	3/12 (25)	6/15 (40)
Ferritin elevation (> 500 µg/l)	134/135 (99)	47/48 (98)	59/59 (100)	13/13 (100)	15/15 (100)
Ferritin peak > 10,000 µg/l	100/135 (74)	37/48 (77)	45/59 (76)	9/13 (69)	9/15 (60)
Ferritin peak > 50,000 µg/l	42/135 (31)	11/48 (23)	24/59 (41)	4/13 (31)	3/15 (20)
Ferritin at initial presentation, Median (µg/l) range	6,747 479–143,210	6,696 479–70,100	11,782 563–143,210	4,175 1,431–15,733	6,373 2,175–50,000
Ferritin peak values, Median (µg/l) range	30,281 479–2,632,220	24,404 479–526,259	39,504 1,254–2,632,220	30,554 1,243–186,833	16,146 3,855–188,620
Soluble CD25 (sIL-2R) (> 2,400 U/ml)	103/109 (94)	40/41 (98)	46/47 (98)	7/11 (64)	10/10 (100)
Median (U/ml) range	7,500 1,194–108,640	11,298 1,194–108,640	7,500 2,125–70,300	5,080 1,333–26,660	7,015 2,472–30,000
Low or absent NK-cell activity	9/21 (43)	2/2 (100)	6/16 (38)	0/2 (0)	1/1 (100)
Low NK-cell count (FACS)	29/44 (66)	7/11 (64)	18/26 (69)	2/4 (50)	2/3 (67)
Hemophagocytosis^+^	81/129 (63)	24/44 (55)	35/57 (61)	8/13 (62)	14/15 (93)
Other					
Elevated alanine aminotransferase (ALAT)	111/131 (85)	38/48 (79)	50/56 (89)	11/13 (85)	12/14 (86)
Elevated ALAT > 2.5 × ULN	75/131 (57)	20/48 (42)	39/56 (70)	8/13 (62)	8/14 (57)
Elevated aspartate aminotransferase (ASAT)	123/133 (92)	43/47 (91)	54/58 (93)	12/13 (92)	14/15 (93)
Elevated ASAT > 2.5 × ULN	98/133 (74)	32/47 (68)	47/58 (81)	8/13 (62)	11/15 (73)
Elevated total bilirubin level	90/132 (68)	33/48 (69)	45/57 (79)	5/13 (38)	7/14 (50)
Elevated total bilirubin level > 2.5 × ULN	59/132 (45)	22/48 (46)	30/57 (53)	3/13 (23)	4/14 (29)
Hypoalbuminemia (< 35 g/l)	109/111 (98)	39/40 (98)	48/49 (98)	8/8 (100)	14/14 (100)
Albumin < 20 g/l	39/111 (35)	15/40 (38)	19/49 (39)	3/8 (38)	2/14 (14)
Elevated lactate dehydrogenase (LDH)	126/133 (95)	45/47 (96)	54/58 (93)	13/13 (100)	14/15 (93)
Elevated LDH > 2.5 × ULN	97/133 (73)	31/47 (66)	46/58 (79)	8/13 (62)	12/15 (80)
Elevated C-reactive protein level	129/132 (98)	46/48 (96)	56/56 (100)	13/13 (100)	14/15 (93)

*M-HLH* malignancy-associated HLH, *I-HLH* infection-associated HLH, *MAS-HLH* macrophage activation syndrome, *ANC* absolute neutrophil count, *sIL-2R * soluble interleukin-2 receptor, *NK-cell* natural killer cell, *FACS* Fluorescence-activated cell sorting, *ULN* upper limit of normal

*One patient had splenectomy prior to HLH diagnosis

^+^Assessed morphologically in either bone marrow, spleen, or lymph node

Functional immune response tests were performed in a proportion of patients. NK-cell degranulation assays were carried out in 21 patients, with pathologic results in 9 (43%). Testing for signaling-lymphocytic-activation-molecule-associated protein (SAP) deficiency was done in 9 patients, a pathologic result was detected in one patient who was diagnosed with X-linked lymphoproliferative disease 1 (XLP-1). Reduced perforin expression was found in three of nine patients who underwent testing (33%), and subsequent genetic analysis in these patients revealed one homozygous (A91V) and one compound-heterozygous mutation (A91V/Q405X) in the perforin gene; in the third patient with reduced perforin expression a heterozygous perforin mutation (A91V) was found. In two more patients, genetic causes were unraveled: Griscelli syndrome type 2 and X-linked lymphoproliferative disease 2 (XLP-2), respectively. Patient characteristics are summarized in Supplementary Table 2.

In addition, targeted sequencing of the perforin gene was performed using blood or bone marrow samples from a total of 20 patients, revealing two patients (10%) carrying heterozygous A91V perforin mutations.

## Treatment

Most patients received anti-inflammatory treatment using glucocorticosteroids (124 of 137 patients, 90.5%), either as monotherapy, in combination with other immunosuppressive agents or as part of chemotherapy regimens such as CHOEP (cyclophosphamide, doxorubicin, vincristine, etoposide, and prednisone) in cases of malignancy-associated HLH. Etoposide was administered in 70 patients (51.1%), intravenous polyvalent immunoglobulins were given in 63 patients (46.0%), and cyclosporine was used in 28 patients (20.4%). In four patients with refractory or relapsing HLH, allogeneic hematopoietic stem cell transplantation was carried out. Rituximab was not only used as part of B-cell lymphoma chemotherapy, but also as a treatment option in EBV infected patients. Of 37 patients, who had more than 2000 EBV copies/ml in whole blood samples (quantitative PCR), 22 (59.5%) received treatment with rituximab. Alemtuzumab (anti-CD52 antibody) or tocilizumab (anti-interleukin-6-receptor antibody) were used in three and two patients, respectively; anakinra, an interleukin-1-receptor antagonist, was applied in selected patients (*n* = 7). In three patients with refractory HLH, cytokine adsorption via column filtration (Cytosorb®) was successfully deployed as salvage therapy.

## Outcome and prognostic factors

**Fig. 1:**
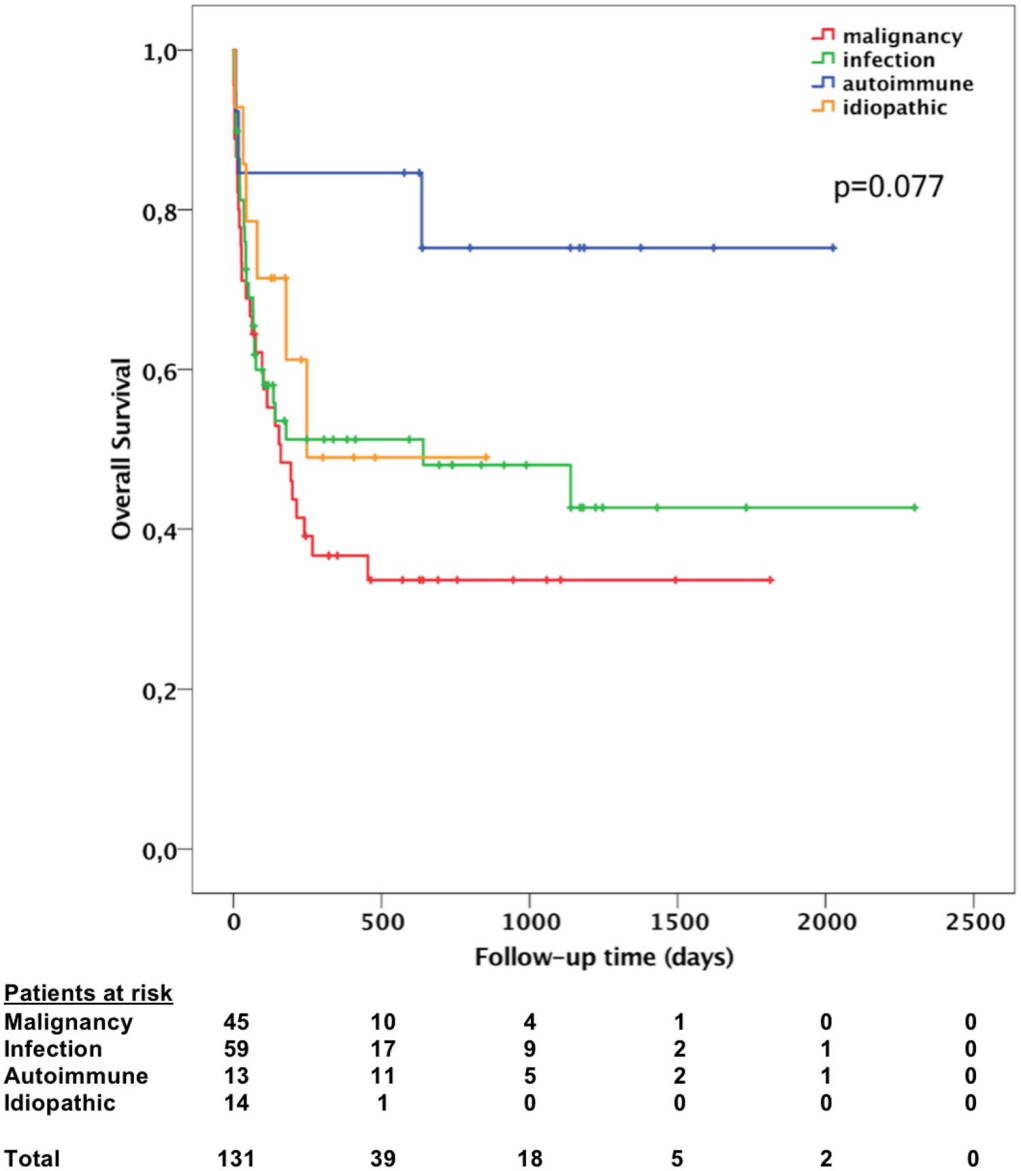
Kaplan–Meier plot showing overall survival for different HLH subgroups. Patients with malignancy-associated HLH had the shortest median survival time, although no statistically significant difference between the subgroups was observed (log-rank test: *p* =  0.077)

In 131 patients follow-up data for survival analysis were available, while six patients were lost to follow-up. Median follow-up time from diagnosis was 154 days, and median overall survival was 267 days. Patients with malignancy-associated HLH had the shortest median survival (160 days), followed by those with idiopathic HLH (248 days), infection-associated HLH (641 days), and MAS-HLH (not reached). However, there was no statistically significant difference between these subgroups (*p* =  0.077 using log-rank test). Figure 1 presents a Kaplan–Meier plot, showing overall survival of different subgroups. Overall, 67 of 131 patients died (51.1%); 27 patients (20.6%) died within 30 days from date of HLH diagnosis. Multiple organ failure was the most common cause of death. To identify possible prognostic factors for both overall and 30-day mortality, univariate and multivariate analyses were conducted. In univariate analysis, age over 50 years, low absolute neutrophil count, hemoglobin below 8.2 g/dl, platelet count below 20*10^9^/l, ferritin concentration above 10,000 µg/l, albumin concentration below 20 g/l, and more than fivefold increased bilirubin concentration were associated with a poor outcome (Table 5). By multivariate analysis, age over 50 years (HR 1.811; 95% CI 1.020–3.217; *p* =  0.043), absolute neutrophil count below 1*10^9^/l (HR 1.861; 95% CI 1.056 – 3.281; *p* =  0.032), platelet count below 20*10^9^/l (HR 2.243; 95% CI 1.210 – 4.158; *p* =  0.010) and albumin concentration below 20 g/l (HR 2.606; 95% CI 1.490–4.561; *p* =  0.001) were independent predictors of poor overall survival (Table 6). Repeating the analysis for death within 30 days after HLH diagnosis, low absolute neutrophil count, platelets below 20*10^9^/l, and albumin below 20 g/l were associated with early death in univariate analysis. Multivariate analysis revealed platelets below 20*10^9^/l (HR 3.446; 95% CI 1.471–8.073; *p* =  0.004) and albumin below 20 g/l (HR 2.531; 95% CI 1.067–6.005; *p* =  0.035) to be independent predictors for early death within 30 days after HLH diagnosis.

**Table 5:** Univariate analysis of possible predictors of mortality

Prognostic factor	Overall			Death within 30 days		
	HR	95% CI	Significance	HR	95% CI	Significance
Age > 50 years	1.994	1.214–3.276	0.006*	1.838	0.841–4.014	0.127
Gender male vs. female	1.032	0.628–1.695	0.902	0.737	0.345–1.574	0.430
Neutrophils < 1*10^9^/l	1.970	1.162–3.341	0.012*	2.615	1.108–6.171	0.028*
Hemoglobin < 8.2 g/dl	1.750	1.070–2.862	0.026*	1.471	0.682–3.170	0.325
Platelets < 20*10^9^/l	2.133	1.270–3.583	0.004*	3.646	1.711–7.768	0.001*
Fibrinogen ≤ 1.5 g/l	1.042	0.631–1.723	0.872	0.633	0.273–1.466	0.286
Ferritin > 10,000 µg/l	2.025	1.034–3.965	0.040*	1.216	0.491–3.012	0.673
Presence of hemophagocytosis	0.674	0.407–1.116	0.125	0.654	0.298–1.433	0.288
Albumin < 20 g/l	2.318	1.386–3.877	0.001*	2.868	1.273–6.461	0.011*
More than fivefold increased Bilirubin	2.272	1.395–3.700	0.001*	1.576	0.741–3.352	0.238

*HR* hazard ratio, *CI* confidence interval

*Indicates statistically significant values

**Table 6:** Multivariate analysis

	Hazard ratio	95% CI	Significance
Overall survival			
Age > 50 years	1.811	1.020–3.217	0.043*
Neutrophils < 1*10^9^/l	1.861	1.056–3.281	0.032*
Hemoglobin < 8.2 g/dl	1.133	0.614–2.090	0.689
Platelets < 20*10^9^/l	2.243	1.210–4.158	0.010*
Ferritin > 10,000 µg/l	1.381	0.645–2.960	0.406
Albumin < 20 g/l	2.606	1.490–4.561	0.001*
More than fivefold increased bilirubin	1.323	0.693–2.526	0.396
Death within 30 days			
Neutrophils < 1*10^9^/l	1.746	0.708–4.304	0.226
Platelets < 20*10^9^/l	3.446	1.471–8.073	0.004*
Albumin < 20 g/l	2.531	1.067–6.005	0.035*

*CI* Confidence interval

*Indicates statistically significant values

## Discussion

Hemophagocytic lymphohistiocytosis (HLH) constitutes a severe hyperinflammatory syndrome emerging from a deregulated immune system due to various triggering conditions. Despite being first described back in 1928 by Tschistowitsch and Bykowa as systemic reticulosis (presumably the first report ever) (Tschistowitsch and Bykowa 1928) and 1939 by Scott and Robb-Smith as histiocytic medullary reticulosis (first report in english) (Bodley Scott and Robb-Smith 1939), HLH in adult patients was neglected for a long time. Increasing awareness developed during the past decade resulting in a growing number of publications (Arca et al. 2015; Bachier Rodriguez and Ritchie 2016; Berliner et al. 2016; Delavigne et al. 2014; Fardet et al. 2010; Halacli et al. 2016; Li et al. 2014, 2015; Machaczka et al. 2011; Otrock and Eby 2015; Park et al. 2012; Ramos-Casals et al. 2014; Riviere et al. 2014; Schram et al. 2016; Tamamyan et al. 2016). However, only few case series on adult HLH are available and essential parts of current management standards for adult patients (i.e., diagnostic criteria, treatment protocols) are adapted from pediatric HLH guidelines. Therefore, a national registry including an associated consulting service was established to collect cases of adult HLH patients and contribute to a better understanding and improved clinical management of this rare and still often fatal syndrome. This registry is part of a campaign to sensitize clinicians in Germany for potential HLH.

HLH in adults emerges from various underlying conditions, as demonstrated in an overview by Ramos-Casals (Ramos-Casals et al. 2014). Considering the data of almost 2200 patients from published case series, infections and malignancies were the most common triggers, followed by autoimmune/autoinflammatory diseases. HLH can also arise during or after chemotherapy, or in the context of organ or stem cell transplantation (Delavigne et al. 2014; Lehmberg et al. 2015).

Of note, underlying conditions such as infections or malignancies alone can lead to the clinical picture of HLH if not adequately controlled; besides, infections may also act as trigger in patients with preexisting autoinflammatory or malignant disorders.

Most recently, reports of cytokine release syndromes due to cellular and bispecific T-cell engaging immunotherapies came into focus, as they share pathophysiologic aspects of HLH and present with a similar clinical phenotype (Lee et al. 2014; Teachey et al. 2013). In our cohort, the triggering entities were in line with the literature, with infections (44.5%) and malignancies (35%) being most frequent. In malignancy-triggered HLH, lymphoma, especially of B-lymphoid origin, was the predominant trigger. This is in contrast to Asian countries, where T-cell lymphoma, in accordance with their higher prevalence, are the most common lymphoma subtype in malignancy associated HLH (Han et al. 2007; La Rosee 2015a; Li et al. 2014).

In infection-associated HLH, viral infections, especially with herpes viruses such as EBV or CMV, were most common. In several patients, bacterial, fungal, or parasitic infections were identified (Table 2). Interestingly, in three patients visceral leishmaniasis was the triggering disease. Two of the affected patients were immunosuppressed by previous treatment with biologicals. In one of three patients, leishmaniasis was diagnosed by polymerase chain reaction (PCR), while bone marrow morphology was negative for intracellular Leishmania amastigotes. Therefore, including leishmania PCR in the diagnostic bone marrow workup is strongly recommended, to safely protect patients from harmful immunosuppression. Specific treatment using liposomal amphotericin is available (Bode et al. 2014). In our registry, one patient was refugee from Albania, and two patients were German tourists returning from Mallorca and Crete. For risk assessment of potential leishmania exposition, World Health Organization (WHO) maps of endemic distribution can be accessed via https://www.who.int. Collaboration with clinical infectiologists is mandatory.

Leishmania-associated HLH demonstrates how important it is to identify the underlying disease, and not to be satisfied with the diagnosis “HLH”. Experience in a French pediatric HLH-series, where three children received etoposide with the diagnosis HLH of unknown origin, and the clinical course of one of our registry patients, who received etoposide after Leishmania was excluded by bone marrow morphology only (without PCR-testing), highlights the risk of treating HLH according to standard protocols without considering specific treatment of the triggering disease. This can also be challenging when lymphomas triggering HLH are masked by inflammatory infiltrates in tissue samples from lymph node, liver, spleen or skin, preventing the pathologist from detecting the malignant tissue component. It is therefore pivotal to carry on sequential diagnostic reassessment by imaging studies, laboratory tests and tissue biopsies along with ongoing treatment. In some cases, splenectomy has been shown to demask lymphomas despite non-informative imaging studies including PET-scan (Jing-Shi et al. 2015).

In general, HLH treatment is based upon three columns: (a) immunosuppression using dexamethasone ± polyvalent immunoglobulins and—if needed—more aggressive immune cell depletion using etoposide, anti-thymocyte globulin or alemtuzumab to interrupt pathologic immune activation, (b) classic measures of intensive care medicine to sustain organ function and prevent severe bleeding, and (c) specific trigger-directed therapy, i.e., chemotherapy, antimicrobial agents or cytokine neutralization. Treatment strategies in adults encompass main components adapted from protocols developed for the control of primary HLH in children (i.e., HLH-94 and HLH-2004 protocol), but are tailored to the individual patient, depending on HLH severity and the underlying trigger (La Rosee 2015b). Upcoming strategies include more specific therapies [i.e., antibodies such as tocilizumab (Teachey et al. 2013), the interferon-gamma antibody emapalumab (Jordan et al. 2015), small molecules such as the Jak1/2-inhibitor ruxolitinib (La Rosee 2016; Sin and Zangardi 2017) or cytokine adsorption, which was successfully used in three of our patients. The latter treatment might be useful to bridge the time from cytokine-dependent organ failure, precluding cytotoxic treatment, to definitive causative treatment (Frimmel et al. 2014; Greil et al. 2017). The intensity of immunosuppression in the pediatric HLH-1994 protocol is neither suitable nor required in the majority of adult HLH patients (Bergsten et al. 2017; Ehl et al. 2018). It seems rather advisable to avoid prolonged immunosuppression, in particular to reduce the risk of subsequent infections. Fever episodes after HLH-directed immunosuppression require careful evaluation for potential secondary infections to avoid harming HLH-salvage treatments.

Primary clinical presentation in our cohort was variable. Besides a triad consisting of fever, hepatosplenomegaly, and cytopenia (62% of our patients), patients presented with renal failure, liver dysfunction (i.e., elevated bilirubin, low albumin, coagulation disorder), neurological symptoms (impaired consciousness, seizures), or bleeding complications. Of note, histological or cytological proof of hemophagocytosis is not necessarily required for diagnosing HLH despite being the eponymous feature. In several studies hemophagocytosis was missing in up to 40% of the patients (Otrock and Eby 2015; Riviere et al. 2014). In our cohort, only 63% of the patients had hemophagocytosis according to assessment of the respective pathologist. On the other hand, hemophagocytosis may also appear in sepsis or rheumatologic disorders, reducing its specificity for HLH (Gupta et al. 2008). Perhaps more rigorous morphological criteria as proposed by Gars et al. might help to differentiate between HLH and other inflammatory conditions. In this study on 78 patients with or without HLH, HLH was strongly associated with hemophagocytosis of granulocytes, nucleated erythrocytes and at least one hemophagocyte engulfing multiple nucleated cells (Gars et al. 2018). Future studies should verify these findings and its feasibility in evaluation of potential HLH.

Diversity of clinical pictures in adult HLH often leads to delayed diagnosis and presumably a high number of unreported cases, as most of the aforementioned symptoms are found in a variety of other diseases (Lachmann et al. 2018). In the analysis of our data, median time to diagnosis was 10 days. However, in about 20% of the patients, time to diagnosis was longer than 3 weeks, highlighting the difficulties in recognizing HLH in due time. Red flags are persisting fever despite broad antibiotic therapy and cytopenia, or a sepsis-like clinical picture, without detected pathogen and only poor response to anti-infective treatment.

Highly elevated ferritin concentrations are a hallmark in HLH. In our study, virtually all patients had elevated ferritin (99%), and 74% of the patients had peak concentrations above 10,000 µg/l, while 31% even had a ferritin peak concentration above 50,000 µg/l. These findings are in accordance with other studies suggesting that ferritin concentrations above 2000 µg/l might be more specific than the threshold used in the HLH-2004 criteria (Fardet et al. 2010; Otrock and Eby 2015; Parikh et al. 2014; Schram et al. 2016). Therefore, extremely high ferritin in the absence of a known iron metabolism disorder, hemolysis or multiple transfusions should lead to evaluation of possible HLH, despite limited specificity in adults (Allen et al. 2008; Otrock et al. 2017; Schram et al. 2015). Besides ferritin, soluble interleukin-2 receptor is a useful diagnostic and monitoring tool, which also might have prognostic significance (Hayden et al. 2017; Zhang et al. 2011b). A high ratio between sIL-2R and ferritin has been suggested as a potential marker for lymphoma-associated HLH and might help in diagnosing patients with yet undetected lymphoma (Lin et al. 2017; Tsuji et al. 2014). Based on the published literature and the experience from our registry we strongly recommend extensive search for possible lymphoma in HLH patients presenting with markedly elevated sIL-2R levels.

In this context, the HScore may be a valuable add-on tool for clinicians if HLH is suspected. The score was developed by a French working group in 2014 to assess the probability of having reactive hemophagocytic syndrome, and to distinguish HLH from other medical conditions such as sepsis (Fardet et al. 2014). Free online availability, graduation of laboratory parameters, and exclusion of elaborate tests, e.g., NK-cell activity, are main advantages of the score. In our cohort, we found a strong correlation between the HScore and HLH-2004 criteria (*r* = 0.75, *p* < 0.001), and 89% of the patients had a probability of more than 90% for HLH according to the HScore (Supplementary Fig. 2). As its calculation is easy and only takes a few minutes, the use of the HScore in addition to HLH-2004 criteria is recommended when evaluating patients with suspected HLH.

NK cell activity was tested in a proportion of our patients. Yet, functional testing (i.e., degranulation or expression assays) may only be advisable in selected patients, i.e., in young male patients with EBV-associated HLH, HLH-relapse or in patients with suspected primary immunodeficiency (i.e., albinism) (Lehmberg and Ehl 2013). If pathologic results are found, a subsequent genetic analysis is suggested, not to miss cases with late-onset hereditary disease. Previous studies demonstrated that mutations in HLH-predisposing genes are present in up to 10% of adult patients with HLH (Wang et al. 2014; Zhang et al. 2011a). In our registry, five patients showed HLH-associated gene mutations—a number possibly underestimating the true value since only a proportion of patients was tested. Besides known disease-causing mutations, monoallelic mutations in HLH-related genes have been described in affected patients, however their impact on HLH development is not fully clear. In a report from the Italian HLH registry, Cetica et al. found monoallelic mutations in 43 of 426 patients analyzed (10.1%), and postulated a potential influence on HLH pathogenesis (Cetica et al. 2016). Of note, the monoallelic perforin A91V mutation occurs in up to 9% of healthy individuals and, as the cumulative incidence of HLH is much lower, cannot be regarded as genetically causative for HLH development (Busiello et al. 2006; Zur Stadt et al. 2004).

Despite advances in treatment strategies, the prognosis in adult HLH is still poor. In previous analyses, HLH mortality rates up to 75% were reported (Parikh et al. 2014; Schram et al. 2016; Shabbir et al. 2011). In our cohort, the overall mortality rate was 51% (67/131). According to various studies, patients with malignancy-associated HLH have the worst prognosis (Machaczka et al. 2011; Otrock and Eby 2015; Parikh et al. 2014; Shabbir et al. 2011; Tamamyan et al. 2016). An overview provided by Daver et al. found a median survival time between 1.2 and 2.4 months for patients with malignancy-associated HLH (Daver et al. 2017). Accordingly, patients with underlying malignancy had the shortest median survival in our cohort, whereas patients with non-malignancy HLH tended to have a better prognosis, although there was no statistical significance (Fig. 1).

Several adverse prognostic factors such as male sex, higher age, malignancy, low platelet counts, or low albumin have been described in adult HLH (Arca et al. 2015; Li et al. 2014; Oto et al. 2015; Otrock and Eby 2015; Parikh et al. 2014). In our study, age above 50 years, neutrophil count less than 1*10^9^/l, platelets under 20*10^9^/l, and albumin under 20 g/l predicted a poor overall survival. These prognostic factors, together with underlying trigger and the extent of laboratory alterations, might help to classify HLH patients into different risk groups. This will allow tailoring HLH-directed and adjusting trigger-directed therapy, i.e., the early use of etoposide in severe HLH cases or consolidation with autologous stem cell transplantation in patients with lymphoma-associated HLH.

Altogether, our study includes a relatively large number of patients from 44 different institutions and thus currently provides the best overview on HLH in adults in Germany. The major limitation arises from retrospective data acquisition and analysis.

HLH in adults is clinically highly heterogeneous with patients presenting in virtually all subdisciplines depending on initial symptoms and etiology. With increasing age, lymphoma becomes the most prevalent HLH-trigger, yet histologic proof of lymphoma often is masked by HLH-associated lymphoproliferation. High suspicion and relentless search for the underlying disease together with interdisciplinary diagnostics and care are cornerstones for reversing the dismal prognosis of HLH. Further cooperative research is necessary to systematically study this rare syndrome and to define optimal treatment algorithms, including novel targeted therapies (i.e., biologic agents).

## Supplementary Information

Below is the link to the electronic supplementary material.

Supplementary file 1 (DOCX 259 KB)

## Appendix: Participating centers

Universitätsklinikum Aachen—J. Panse, B. Voss, D. Goy.

Klinikum Augsburg—K. Hirschbühl.

Charité – Universitätsmedizin Berlin, Campus Virchow-Klinikum—O. Penack.

Vivantes Auguste-Viktoria-Klinikum Berlin—M. Müller, H. Stocker.

Evangelisches Klinikum Bethel, Bielefeld—F. Weißinger.

Medizinische Universitätsklinik Knappschaftskrankenhaus Bochum—R. Schroers.

Klinikum Chemnitz—R. Herbst, A. Morgner.

Krankenhaus Dornbirn—W. Bair.

Klinikum Dortmund—M. Unnewehr.

HELIOS Klinikum Erfurt—H. Sayer.

Universitätsklinikum Erlangen—S. Krause, E. Hofmann.

Klinikum Esslingen—M. Geißler.

Klinikum Frankfurt Höchst—F. Scholten.

Universitätsklinikum Freiburg—S. Ehl, F. Röther.

Universitätsklinikum Gießen—F. Ohliger, J. Trauth.

Universitätsmedizin Göttingen—D. Burghardt.

Universitätsmedizin Greifswald—M. Lerch, C. Hirt.

Universitätsklinikum Halle (Saale)—T. Weber.

Universitätsklinikum Hamburg-Eppendorf—S. Schliffke, Christina Dicke.

Klinikum Heidenheim—M. Müller.

Universitätsklinikum Schleswig–Holstein Kiel—M. Ritgen, D. Schewe.

Universitätsklinikum Köln—S. Krämer.

St. Marien- und St. Annastiftskrankenhaus Ludwigshafen—P. Meier.

Universitätsmedizin Mainz—P. Wölfinger.

Städtisches Klinikum München Schwabing—M. Starck.

Klinikum der Universität München, Standort Großhadern—M. Subklewe, V. Bücklein.

Rotkreuzklinikum München—M. Hentrich.

Gemeinschaftspraxis für Hämatologie und Onkologie Münster—R. Liersch.

Universitätsklinikum Münster—B. Baumgarten, A. Schulze, M. Mohr.

Klinikum Nürnberg Nord—S. Dressler.

Klinikum Ernst von Bergmann Potsdam—G. Maschmeyer, F. Rothmann.

Krankenhaus Barmherzige Brüder Regensburg—J. Braess.

Universitätsklinikum Regensburg—T. Lange, M. Grube, M. Fante.

Universitätsmedizin Rostock—D. Gläser, J. Lakner, M. Bärenklau.

Klinikum Sindelfingen-Böblingen—M. Ritter.

Universitätsklinikum Tübingen—R. Riessen, M. Haap.

Universitätsklinikum Ulm – A. Viardot.

Schwarzwald-Baar Klinikum Villingen-Schwenningen—M. Henkes.

Klinikum Wels-Grieskirchen—S. Burgstaller.

HELIOS Dr. Horst Schmidt Kliniken Wiesbaden—N. Frickhofen, B. Jung.

Rems-Murr-Kliniken Winnenden—S. Parmentier.

Klinikum Wolfsburg—S. Neumann.

Heinrich-Braun-Klinikum Zwickau—S. Graupner.
