# Supplementary material for: Hemophagocytic lymphohistiocytosis in adults: collaborative analysis of 137 cases of a nationwide German registry
Source: J Cancer Res Clin Oncol. 2020 Feb 20;146(4):1065–77. doi: 10.1007/s00432-020-03139-4 (PMC7085479; doi:10.1007/s00432-020-03139-4)

**Supplementary information**

**Hemophagocytic lymphohistiocytosis in adults: Collaborative analysis of 137 cases of a nationwide German registry**

Sebastian Birndt^1^, Thomas Schenk^1^, Babett Heinevetter^1^, Frank M. Brunkhorst^2^, Georg Maschmeyer^3^, Frank Rothmann^3^, Thomas Weber^4^, Markus Müller^5^, Jens Panse^6^, Olaf Penack^7^, Roland Schroers^8^, Jan Braess^9^, Norbert Frickhofen^10^, Stephan Ehl^11^, Gritta Janka^12^, Kai Lehmberg^12^, Mathias W. Pletz^13^, Andreas Hochhaus^1^, Thomas Ernst^1^, Paul La Rosée^14^

*^1^Klinik für Innere Medizin II, Abt. Hämatologie und intern. Onkologie, Universitätsklinikum Jena, Germany;*

*^2^Zentrum für klinische Studien, Universitätsklinikum Jena, Germany;*

*^3^Klinik für Hämatologie, Onkologie u. Palliativmedizin, Klinikum Ernst von Bergmann, Potsdam, Germany;*

*^4^Klinik für Hämatologie und Onkologie, Universitätsklinikum Halle (Saale), Germany;*

*^5^Zentrum für Infektiologie und HIV, Vivantes Auguste-Viktoria-Klinikum, Berlin, Germany;*

*^6^Klinik für Hämatologie, Onkologie, Hämostaseologie und Stammzelltransplantation, Uniklinik RWTH Aachen, Germany;*

*^7^ Medizinische Klinik mit Schwerpunkt Hämatologie, Onkologie und Tumorimmunologie, Charité Universitätsmedizin, Berlin, Germany;*

*^8^Hämatologie und Onkologie, Universitätsklinikum Knappschaftskrankenhaus, Bochum, Germany;*

*^9^Onkologie und Hämatologie, Krankenhaus Barmherzige Brüder, Regensburg, Germany;*

*^10^Hämatologie, Onkologie und Palliativmedizin, HELIOS Dr. Horst Schmidt Kliniken, Wiesbaden, Germany;*

*^11^ Institut für Immundefizienz, Zentrum für Chronische Immundefizienz, Universitätsklinikum Freiburg, Germany;*

*^12^Pädiatrische Hämatologie und Onkologie, Universitätsklinikum Eppendorf, Hamburg, Germany;*

*^13^Institut für Infektionsmedizin und Krankenhaushygiene, Universitätsklinikum Jena, Germany;*

*^14^Klinik für Innere Medizin II, Hämatologie, Onkologie, Immunologie, Infektiologie und Palliativmedizin, Schwarzwald-Baar Klinikum, Villingen-Schwenningen, Germany*

**Supplementary Table 1.**

**Calculation of the HScore, adapted from Fardet et al.^3^**

| **Parameter** | **Number of points (criteria for scoring)** |
| --- | --- |
| Known underlying immunosuppression* | 0 (no) or 18 (yes) |
| Temperature (°C) | 0 (<38.4), 33 (38.4 – 39.4), or 49 (>39.4) |
| Organomegaly | 0 (no), 23 (hepatomegaly or splenomegaly), or 38 (hepatomegaly and splenomegaly) |
| Type of cytopenias^+^ | 0 (1 lineage), 24 (2 lineages), or 34 (3 lineages) |
| Ferritin (µg/liter) | 0 (<2,000), 35 (2,000 – 6,000), or 50 (>6,000) |
| Triglycerides (mmol/liter) | 0 (<1.5), 44 (1.5 – 4), or 64 (>4) |
| Fibrinogen (g/liter) | 0 (>2.5) or 30 (≤2.5) |
| Serum aspartate aminotransferase (ASAT) (IU/liter) | 0 (<30) or 19 (≥30) |
| Hemophagocytosis features on bone marrow aspirate | 0 (no) or 35 (yes) |

* Human immunodeficiency virus positive or receiving ling-term immunosuppressive therapy (i.e. glucocorticoids, cyclosporine, azathioprine)

^+^ Defined as a hemoglobin level of ≤9.2 g/dl and/or a leukocyte count of ≤5,000/mm^3^ and/or a platelet count of ≤110,000/mm^3^

**Supplementary Table 2.**

**Information on patients with HLH-associated mutations.**

| Patient | Sex | Age at diagnosis | Triggering disease | Mutated gene |
| --- | --- | --- | --- | --- |
| 1 | Male | 23 | CMV/EBV co-infection | XIAP (XLP-2) |
| 2 | Male | 19 | EBV/H1N1 infection | SH2D1A (XLP-1) |
| 3 | Female | 43 | NK/T-cell lymphoma | PRF1 (A91V/Q405X compound heterozygous) |
| 4 | Male | 51 | Idiopathic | PRF1 (A91V homozygous) |
| 5 | Female | 24 | EBV infection | RAB27A (Griscelli syndrome type 2) |

Abbreviations: EBV, Epstein-Barr virus; CMV, Cytomegalovirus; XLP, X-linked lymphoproliferative disease; XIAP, X-linked inhibitor of apoptosis protein; SH2D1A, SH2 domain-containing protein 1A; PRF, Perforin; RAB27A, Ras-related protein Rab-27A.

**Figure legends**

**Supplementary Figure 1.**

Flow chart illustrating the enrollment process and eligibility for data analysis.

**Supplementary Figure 2.**

Scatter plot illustrating the relationship between HLH-2004 diagnostic criteria and the HScore. Using Pearson´s r, there was a strong positive correlation between number of fulfilled diagnostic criteria and the HScore (r=0.75, p<0.001).

**Supplementary Figure 1.**


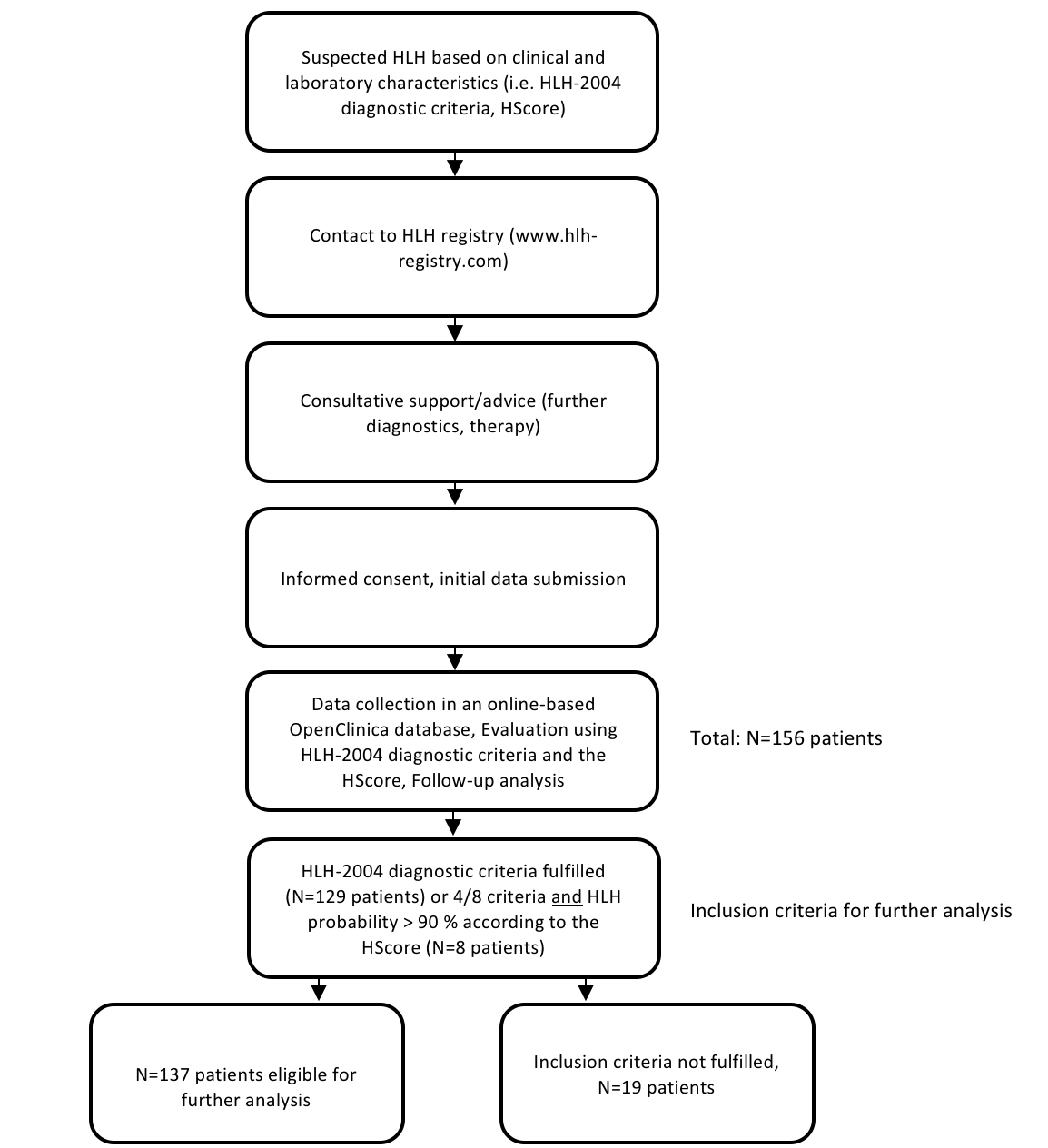


**Supplementary Figure 2.**


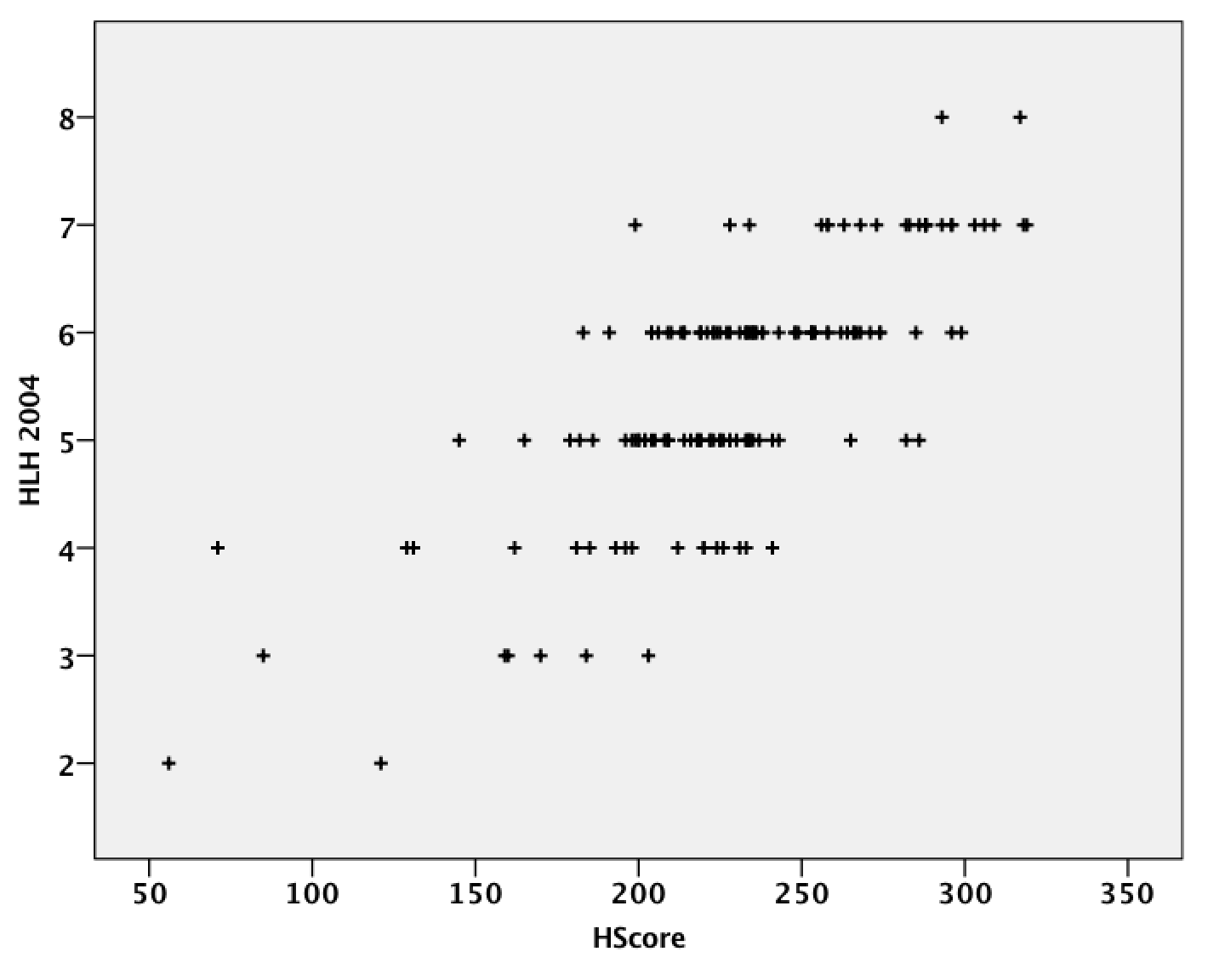

Supplement: Supplementary file 1 — (DOCX 259 KB) [file 432_2020_3139_MOESM1_ESM.docx]
